# Supplementary material for: CurriMAE: curriculum learning based masked autoencoders for multi-labeled pediatric thoracic disease classification
Source: PeerJ Comput Sci. 2025 Jul 9;11:e3019. doi: 10.7717/peerj-cs.3019 (PMC12453726; doi:10.7717/peerj-cs.3019)
Supplement: Supplemental Information 1 [file peerj-cs-11-3019-s001.docx]

**CurriMAE: Curriculum learning based masked autoencoders for multi-labeled pediatric thoracic disease classification**

Taeyoung Yoon, Daesung Kang

School of Bio-Health Convergence, College of Natural Sciences, Sungshin Women’s University Woonjung Green Campus, Seoul, Republic of Korea

Table S1. Environmental setup for model training and evaluation.

| Component | Specification |
| --- | --- |
| Operating System | Ubuntu Linux 24.04 |
| CPU | Intel Core Ultra 7 265KF (Arrow Lake) |
| RAM | 128 GB |
| GPU | NVIDIA GeForce RTX 5090 SOLID OC (32 GB VRAM) |
| CUDA version | 12.8 |
| PyTorch version | 2.7.0 |
| Timm library | 0.3.2 |

Table S2. CurriMAE performance across different learning rates (highest performance values in bold; standard deviations in parentheses).

| Learning rate | AUC | Sensitivity | Precision | F1-score |
| --- | --- | --- | --- | --- |
| 1e-3 | 0.736 (0.010) | 0.738 (0.016) | 0.533 (0.010) | 0.608 (0.008) |
| 2.5e-3 | **0.755 (0.003)** | **0.759 (0.012)** | **0.552 (0.010)** | **0.622 (0.001)** |
| 1e-4 | 0.703 (0.010) | 0.716 (0.006) | 0.522 (0.008) | 0.583 (0.005) |

Table S3. CurriMAE performance across different beta weights.

| Beta weights | AUC | Sensitivity | Precision | F1 Score |
| --- | --- | --- | --- | --- |
| $\beta_{1}=0.9, \beta_{2}=0.99$ | 0.755 (0.003) | 0.759 (0.012) | 0.552 (0.010) | 0.622 (0.001) |
| $\beta_{1}=0.9, \beta_{2}=0.999$ | 0.754 (0.004) | 0.758 (0.007) | 0.548 (0.009) | 0.621 (0.004) |

Table S4. CurriMAE performance across different pretraining epochs (Highest performance values in bold; standard deviations in parentheses).

| Epochs | AUC | Sensitivity | Precision | F1 Score |
| --- | --- | --- | --- | --- |
| 400 epochs | 0.742 (0.004) | 0.751 (0.002) | 0.545 (0.005) | 0.613 (0.005) |
| 800 epochs | **0.755 (0.003)** | **0.759 (0.012)** | **0.552 (0.010)** | **0.622 (0.001)** |
